# Performance of oral HPV DNA, oral HPV mRNA and circulating tumor HPV DNA in the detection of HPV‐related oropharyngeal cancer and cancer of unknown primary

**DOI:** 10.1002/ijc.33798

**Published:** 2021-09-20

**Authors:** Hidenori Tanaka, Motoyuki Suzuki, Norihiko Takemoto, Takahito Fukusumi, Hirotaka Eguchi, Erina Takai, Haruka Kanai, Mitsuaki Tatsumi, Masafumi Horie, Yukinori Takenaka, Shinichi Yachida, Hidenori Inohara

**Affiliations:** ^1^ Department of Otorhinolaryngology‐Head and Neck Surgery Osaka University Graduate School of Medicine Suita Osaka Japan; ^2^ Department of Cancer Genome Informatics Osaka University Graduate School of Medicine Suita Osaka Japan; ^3^ Department of Nuclear Medicine and Tracer Kinetics Osaka University Graduate School of Medicine Suita Osaka Japan; ^4^ Integrated Frontier Research for Medical Science Division, Institute for Open and Transdisciplinary Research Initiatives (OTRI) Osaka University Suita Osaka Japan; ^5^ Division of Genomic Medicine National Cancer Center Institute Tokyo Japan

**Keywords:** cancer of unknown primary, circulating tumor DNA, droplet digital PCR, human papillomavirus, oropharyngeal cancer

## Abstract

A biomarker that is useful for the detection of human papillomavirus (HPV)‐related oropharyngeal cancer (OPC) and cancer of unknown primary (CUP) is indispensable. We evaluated the diagnostic performance of HPV DNA and mRNA in oral gargle samples and circulating tumor HPV16 DNA (ctHPV16DNA) in blood samples. Oral HPV DNA and mRNA were analyzed using commercially available HPV assays of the GENOSEARCH HPV31 and Aptima, respectively. ctHPV16DNA was analyzed using in‐house droplet digital polymerase chain reaction. Seventy‐four patients with OPC and eight patients with CUP were included. The sensitivity and specificity of oral HPV DNA, oral HPV mRNA, and ctHPV16DNA were 82% (95% confidence interval [CI] = 66‐92) and 100% (95% CI = 88‐100), 85% (95% CI = 69‐94) and 94% (95% CI = 73‐100), and 93% (95% CI = 81‐99) and 97% (95% CI = 84‐100), respectively, for HPV16‐related OPC, while those were 20% (95% CI = 1‐72) and 100% (95% CI = 3‐100), 0% (95% CI = 0‐52) and 100% (95% CI = 3‐100), and 100% (95% CI = 54‐100) and 100% (95% CI = 16‐100), respectively, for HPV16‐related CUP. The sensitivity of ctHPV16DNA for HPV16‐related OPC was higher than that of oral biomarkers, though the difference was not statistically significant. ctHPV16DNA remarkably correlated with the anatomic extent of disease, total metabolic tumor volume and HPV16 copy number per tumor genome in patients with HPV16‐related OPC/CUP, whereas oral biomarkers did not. In conclusion, ctHPV16DNA is a potentially promising biomarker for HPV16‐related OPC, while further studies are required for HPV16‐related CUP.

## Abstract 2

What's new?

A minimally‐invasive biomarker that allows the detection of human papillomavirus (HPV)‐related oropharyngeal cancer and cancer of unknown primary is indispensable. Here, the authors show that circulating tumour HPV DNA (ctHPVDNA) correlates with the tumour burden and HPV copy number per tumour genome in HPV‐related oropharyngeal cancer and cancer of unknown primary. Neither oral HPV DNA nor mRNA exhibits such a correlation. ctHPVDNA outperforms oral HPV DNA and mRNA in detecting HPV‐related oropharyngeal cancer and in distinguishing HPV‐related cancer of unknown primary from HPV‐unrelated cancers. ctHPVDNA emerges as a potentially useful biomarker for HPV‐related oropharyngeal cancer and cancer of unknown primary.

**Abbreviations**
ctHPV16DNA: circulating tumor human papillomavirus 16 DNA
ctHPVDNA: circulating tumor human papillomavirus DNA
ddPCR: droplet digital PCR
FDG‐PET/CT: ^18^F‐fluorodeoxyglucose‐positron emission tomography/computed tomography
HPV: human papillomavirus
MTV: metabolic tumor volume
OPSCC: oropharyngeal squamous cell carcinoma
PCR: polymerase chain reaction
SCCUP: squamous cell carcinoma of unknown primary
SUV: standardized uptake value
UICC: Union for International Cancer Control

## 1 INTRODUCTION

High‐risk human papillomavirus (HPV) infects the epithelium of the oropharynx and promotes oncogenic transformation, leading to the development of HPV‐related oropharyngeal squamous cell carcinoma (OPSCC). Among high‐risk HPVs, HPV16 is a predominant driver in the pathogenesis of HPV‐related OPSCC.^1^ Tumor HPV DNA‐positivity indicates that the tumor is relevant to HPV infection, while it does not always endorse that the tumor is caused by HPV infection. Overexpression of p16 by immunohistochemistry in tumor cells is a surrogate marker of HPV‐driven transformation, although approximately 10% of the p16‐positive OPSCC lacks HPV DNA‐positivity.^2^ OPSCC that is positive for both p16 and HPV DNA is regarded as “truly” HPV‐related, while OPSCC that is positive for either p16 or HPV DNA alone is not regarded as “truly” HPV‐related.^3^ Survival of patients with OPSCC differs significantly according to tumor p16 and HPV DNA status.^4^, ^5^ Patients with p16‐positive/HPV DNA‐positive OPSCC survive significantly longer than patients with p16‐positive/HPV DNA‐negative OPSCC. Patients with p16‐negative/HPV DNA‐positive OPSCC or p16‐negative/HPV DNA‐negative OPSCC have the shortest survival. The incidence of HPV‐related OPSCC continues to increase worldwide,^6^, ^7^ while the natural history from HPV infection to OPSCC development remains largely unknown.^8^ For example, HPV‐related precancerous lesions of the oropharynx have never been identified. When patients with HPV‐related node‐positive squamous cell carcinoma of unknown primary (SCCUP) undergo tonsillectomy, the occult primary that is positive for both p16 and HPV DNA is often identified in the ipsilateral tonsil,^9^, ^10^ suggesting that HPV‐related SCCUP may actually be HPV‐related OPSCC in a yet unknown proportion of cases. Moreover, tumor HPV status is associated with survival in patients with SCCUP as in patients with OPSCC.^11^ The definition of p16‐positive SCCUP has been newly incorporated into the eighth edition of the Union for International Cancer Control (UICC) TNM classification system, although an invasive diagnostic modality, such as open biopsy of the neck, is necessary to prove tumor p16‐positivity. Collectively, a less invasive biomarker that is useful for both the early detection of HPV‐related OPSCC and the differentiation of HPV‐related node‐positive SCCUP from their HPV‐unrelated counterparts is deemed indispensable.

Liquid biopsies that analyze tumor materials, such as cells or nucleic acids, which are obtained in a minimally invasive or noninvasive manner from the blood or other body fluids, have emerged as novel modalities for cancer management.^12^ In patients with HPV‐related OPSCC, oral HPV DNA or mRNA in the saliva and cell‐free circulating tumor HPV DNA (ctHPVDNA) in the plasma represent candidates for a noninvasive biomarker with applicability in early diagnosis and screening. We aimed to evaluate the performance of these biomarkers for detecting HPV‐related OPSCC/SCCUP. Sensitivity and specificity were explored among individuals with newly diagnosed HPV‐related or HPV‐unrelated OPSCC/SCCUP. We also correlated these biomarkers with the extent of the disease. This report is the first to analyze oral HPV DNA, oral HPV mRNA and ctHPVDNA in the same individuals.

## 2 MATERIALS AND METHODS

### 2.1 Patients and definition of HPV‐related tumor

Patients with newly diagnosed OPSCC or node‐positive SCCUP, who first visited the Osaka University Hospital between 2017 and 2020, were prospectively enrolled in the study. Pathological diagnosis was based on a biopsy of the primary site for OPSCC and on fine needle aspiration or open biopsy of the neck for SCCUP. Biopsy specimens of OPSCC were examined to ascertain p16 and HPV DNA status, as detailed below. Fine needle aspiration specimens of SCCUP were also assessed to determine HPV DNA status. p16 status of SCCUP was assessed only when surgically excised node specimens were available. OPSCC that was positive for both p16 and any high‐risk HPV DNA and SCCUP that was positive for any high‐risk HPV DNA were defined as HPV‐related. Likewise, OPSCC that was positive for both p16 and HPV16 DNA and SCCUP that was positive for HPV16 DNA was defined as HPV16‐related. OPSCC and SCCUP that were related to high‐risk HPV except for HPV16 were defined in the same manner. Overexpression of p16 was not considered for the definition of HPV‐related SCCUP. This is because we previously found that SCCUP with metastatic node that was high‐risk HPV DNA‐positive was also always p16‐positive.^9^ High‐risk HPV DNA‐positivity in metastatic nodes alone would be sufficient to define SCCUP as “truly” HPV‐related. Tumors were staged according to the seventh and eighth editions of the UICC TNM classification system. Although, according to the eighth edition, SCCUP with p16‐positive metastatic node has been newly defined as HPV‐related SCCUP, herein, we defined SCCUP harboring high‐risk HPV DNA‐positive metastatic nodes as such even without the evidence of p16‐positivity.

### 2.2 Assessment of tumor p16 and HPV DNA status

DNA was extracted from fresh frozen biopsy or fine needle aspiration specimens using the DNeasy Blood & Tissue Kit (Qiagen, Hilden, Germany) or QIAamp DNA Mini Kit (Qiagen). Extracted DNA was used for nested polymerase chain reaction (PCR) followed by direct sequencing to detect and genotype HPV, as per previously reported methods.^9^ Briefly, nested PCR involved primary PCR using the PGMY09/11 primer set and secondary PCR using the GP5+/6+ primer set. The secondary PCR products were purified and sequenced directly using a genetic analyzer. Typing was achieved by comparing the sequences with those of known HPV types using the National Center for Biotechnology Information Basic Local Alignment Search Tool program (http://blast.ncbi.nlm.nih.gov/Blast.cgi). HPV genotypes of 16, 18, 31, 33, 35, 39, 45, 51, 52, 56, 58, 59, 68 and 69 were classified as high‐risk. Formalin‐fixed paraffin‐embedded biopsy or surgically excised specimen sections were immunohistochemically examined to ascertain p16 status using the antibody clone p16 CINtec E6H4 (Roche, Basel, Switzerland). p16 was scored positive if ≥70% of the tumor cells exhibited robust and diffuse nuclear and cytoplasmic staining results.

### 2.3 Sample collection

Both oral and blood samples were collected prior to any treatment. Participants were advised to gargle with 10 mL of phosphate‐buffered saline for 30 seconds. After gargling, expectorated fluid samples (oral sample) were obtained using a sterile collection tube. A volume of 2 mL of the oral sample was transferred to an Aptima Urine Specimen Transport Tube (Hologic, Inc., San Diego, CA). Both oral samples remaining in the collection tubes and those transferred to the Aptima Urine Specimen Transport Tube were immediately shipped at 4°C to the LSI Medience Corporation (Tokyo, Japan) and stored at 4°C until HPV DNA and mRNA testing. Blood samples (8.5 mL) were collected using a Cell‐Free DNA Collection Tube (Roche). After centrifugation at 3500 rpm for 10 minutes, the plasma was retrieved and stored at −80°C until use. The process from blood collection to storage was completed within 2 to 3 hours.

### 2.4 Assessment of oral HPV DNA and mRNA

For the assessment of oral HPV DNA, DNA was extracted from a 400 μL aliquot of the oral sample in the collection tube using the QIAsymphony SP/AS Instruments (Qiagen). Extracted DNA was then eluted in a final volume of 60 μL and was analyzed using the GENOSEARCH HPV31 kit (Medical and Biological Laboratory, Nagoya, Japan) to detect and genotype HPV. The GENOSEARCH HPV31 kit allows the identification of 31 HPV genotypes, including high‐risk types (16, 18, 31, 33, 35, 39, 45, 51, 52, 56, 58, 59 and 68) and low‐risk types (6, 11, 26, 42, 44, 53, 54, 55, 61, 62, 66, 70, 71, 73, 82, 84, 90 and CP6108), using reverse sequence‐specific oligonucleotide PCR. Briefly, DNA samples were amplified using multiplex PCR. The resultant amplicon mixtures were hybridized with genotype‐specific probes, which were detected using the Luminex System (Luminex, Austin, TX). β‐globin was used as an internal control to verify the presence of sufficient cellular components. For the assessment of oral HPV mRNA, the Aptima HPV assay kit (Hologic) that allows the detection of E6/E7 mRNA from 13 high‐risk HPVs (HPV16, 18, 31, 33, 35, 39, 45, 51, 52, 56, 58, 59 and 68) and HPV66 without providing genotype‐specific information was used. The Aptima HPV assay includes a control for monitoring the entire process. Aliquots (2 mL) of the oral samples in the Aptima Urine Specimen Transport Tube were automatically analyzed using the Panther system (Hologic) according to the manufacturer's instructions. Assessment of oral HPV DNA and mRNA was completed within a week after sample collection.

### 2.5 Quantification of circulating tumor HPV16 DNA

Cell‐free DNA was extracted from 3 mL of cryopreserved plasma and was suspended in 100 μL using the QIAamp Circulating Nucleic Acid Kit (Qiagen). The amount was measured using the Qubit 2.0 fluorometer and Qubit dsDNA HS Assay Kit (Invitrogen, Carlsbad, CA). HPV16 E6 and E7 copy numbers were quantified through the use of our in‐house optimized droplet digital PCR (ddPCR) using the QX200 Droplet Digital PCR System (Bio‐Rad, Hercules, CA), as per methods reported previously.^13^ Briefly, each 20 μL reaction assay comprised 10 μL of 2× ddPCR Supermix for Probes (no dUTP) (Bio‐Rad), 1 μM of each forward and reverse primer, 250 nM of probe and 2 μL of cell‐free DNA. After emulsification using the QX200 Droplet Generator (Bio‐Rad), PCR was performed using the T100 Thermal Cycler (Bio‐Rad) at 95°C for 10 minutes, followed by 40 cycles of two‐step PCR with denaturation at 94°C for 15 seconds and annealing for 60 seconds. The absorbance of the droplet was measured using the QX200 Droplet Reader (Bio‐Rad), and the results were analyzed using the QuantaSoft software v1.7.4.0917 (Bio‐Rad). The number of circulating tumor HPV16 DNA (ctHPV16DNA) copies per mL of plasma was defined as the average of the number of E6 and E7 copies per mL of plasma.

### 2.6 Quantification of HPV16 copy number per tumor genome

To evaluate HPV16 copy number per tumor genome, genomic DNA extracted from fresh frozen biopsy or fine needle aspiration specimens was analyzed with quantitative PCR using the HPV Genotypes 14 Real‐TM Quant kit (Sacace Biotechnologies, Como, Italy) and Bio‐Rad CFX96 qPCR instrument (Bio‐Rad) according to the manufacturer's instructions. HPV16 copy number per tumor genome was calculated with reference to the β‐globin copy number.

### 2.7 Measurement of metabolic tumor volume

Metabolic tumor volume (MTV) was measured from pretreatment attenuation‐corrected ^18^F‐fluorodeoxyglucose‐positron emission tomography/computed tomography (FDG‐PET/CT) data with a standardized uptake value (SUV) threshold of 2.5, using PETSTAT Viewer Version 2.2 (Adln Research Inc., Tokyo, Japan), as per methods reported previously.^14^, ^15^ Total MTV was defined as the sum of MTVs of the tumor at any location, including local, regional and distant sites.

### 2.8 Statistical analysis

The sensitivity of oral HPV DNA for HPV‐related tumor was estimated, on the presupposition that the genotype of oral HPV DNA had to be consistent with that of tumor HPV DNA so that the test results were considered positive. The specificity of oral HPV DNA for any high‐risk HPV‐related tumor was calculated as the number of patients who had negative test results for any high‐risk HPV out of patients with HPV‐unrelated tumor. The specificity oral HPV16 DNA for HPV16‐related tumor was calculated as the number of patients who had negative test results for HPV16 out of patients with HPV16‐unrelated tumor, including HPV‐unrelated tumor and tumor that was related to high‐risk HPV except for HPV16. The specificity of oral HPV DNA for tumor that was related to high‐risk HPV except for HPV16 was calculated as the number of patients who had negative test results for high‐risk HPV except for HPV16 out of patients with HPV‐unrelated tumor because otherwise the specificity had to be calculated for each high‐risk HPV genotype. The sensitivity of oral HPV mRNA for HPV‐related tumor was calculated as the number of patients who had positive test results out of patients with HPV‐related tumor, while the specificity was calculated as the number of patients who had negative test results out of patients with HPV‐unrelated tumor. This is because HPV genotype was unidentifiable with the oral HPV mRNA test. The sensitivity and specificity of ctHPV16DNA for HPV16‐related tumor was calculated in the same manner as those of oral HPV DNA for HPV16‐related tumor. We calculated 95% confidence interval (CI) for sensitivity and specificity with the ClopperPearson method. The Fisher's exact test was used to compare categorical variables, while the exact Wilcoxon rank‐sum test or Kruskal‐Wallis test was used to compare continuous variables, as appropriate. Spearman's rank correlation was used to assess the relationship between two continuous variables. Trends were evaluated using the Jonckheere‐Terpstra test. All statistical analyses were performed using JMP Pro 14 (SAS Institute, Cary, NC) or IBM SPSS Statistics 26 (IBM, Armonk, NY), and statistical significance was set at *P* < .05.

## 3 RESULTS

### 3.1 Study population

We had a total of 100 and 14 patients with newly diagnosed OPSCC and node‐positive SCCUP, respectively, between 2017 and 2020 at Osaka University Hospital. As shown in Figure S1, 74 patients with OPSCC (53 HPV‐related and 21 HPV‐unrelated) and eight patients with node‐positive SCCUP (seven HPV‐related and one HPV‐unrelated) were finally included in the study. Of the 53 patients with HPV‐related OPSCC, 42 had HPV16‐related tumors and 11 had tumors that were related to high‐risk HPV except for HPV16. Of the seven patients with HPV‐related SCCUP, six and one had tumors that were genotyped as HPV16 and HPV33, respectively. Three of the seven patients with HPV‐related SCCUP underwent an open biopsy of the neck node, which revealed p16‐positivity on immunohistochemical analysis. Thus, p16 status was unknown in four of the seven patients with HPV‐related SCCUP. Paired oral and blood samples were available for 68 patients with OPSCC and six patients with SCCUP, while six patients with OPSCC and two patients with SCCUP provided only blood samples. Table 1 shows the baseline characteristics of patients with HPV16‐related OPSCC or SCCUP who underwent the oral or plasma HPV test, while those of patients with OPSCC or SCCUP that was related to high‐risk HPV except for HPV16 are shown in Table S1. Baseline characteristics of patients with HPV‐unrelated OPSCC or SCCUP are shown in Table S2. A tumor that was p16‐negative/high‐risk HPV DNA‐positive was not observed in any patient, and none of the patients presented with tumors that were positive for low‐risk HPV DNA. Of note, one female patient with HPV‐related OPSCC and one female patient with HPV‐related SCCUP had a history of cervical cancer. Both patients had been free from the disease for more than 10 years when they were diagnosed as having OPSCC/SCCUP. No other patients had a history of cervical or anogenital cancer.

**TABLE 1:** Baseline characteristics of patients with HPV16‐related OPSCC or SCCUP

	HPV16‐related OPSCC		HPV16‐related SCCUP	
	Oral sample, N = 39	Blood sample, N = 42	Oral sample, N = 5	Blood sample, N = 6
*Sex*				
Male	30	33	4	5
Female	9	9	1	1
*Age*				
Median	69	69	55	58
Range	43‐93	43‐90	53‐68	53‐68
*Smoking history*				
<10 pack‐years	13	13	2	2
≥10 pack‐years	26	29	3	4
*p16 IHC/HPV DNA status*				
p16‐positive/HPV16‐positive	39	42	1	2
p16‐unknown/HPV16‐positive	0	0	4	4
*Primary subsite*				
Lateral wall	31	32	—	—
Anterior wall	7	9	—	—
Posterior wall	1	1	—	—
Unknown	—	—	5	6
*T classification*				
T0/T1/T2/T3/T4	‐/6/24/4/5	‐/6/24/4/8	5/‐/‐/‐/‐	6/‐/‐/‐/‐
*N classification* ^a^				
N0/N1/N2a/N2b/N2c/N3	6/10/2/13/6/2	6/10/2/13/9/2	0/0/1/2/2/0	0/0/1/2/2/1
*N classification* ^b^				
N0/N1/N2/N3	6/25/6/2	6/25/9/2	0/3/2/0	0/3/2/1
*M classification*				
M0/M1	39/0	41/1	5/0	5/1
*Stage* ^a^				
I/II/III/IV	2/3/10/24	2/3/10/27	0/0/0/5	0/0/0/6
*Stage* ^b^				
I/II/III/IV	26/6/7/0	26/6/9/1	0/3/2/0	3/2/0/1
*Total MTV*				
Median	25.4	28.9	21.9	28.1
Range	0‐255.7	0‐255.7	15.2‐47.5	15.2‐301.6
*HPV16 copy number per tumor genome*				
Median	40.7	39.8	15.9	46.7
Range	0.03‐1380.4	0.03‐1380.4	2.1‐83.2	2.1‐1258.9

Abbreviations: HPV, human papillomavirus; IHC, immunohistochemistry; MTV, metabolic tumor volume; OPSCC, oropharyngeal squamous cell carcinoma; SCCUP, squamous cell carcinoma of unknown primary; UICC, Union for International Cancer Control.

^a^ According to the seventh edition of UICC TNM classification system.

^b^ According to the eighth edition of UICC TNM classification system.

### 3.2 Performance of ctHPV16DNA

**FIGURE 1:**
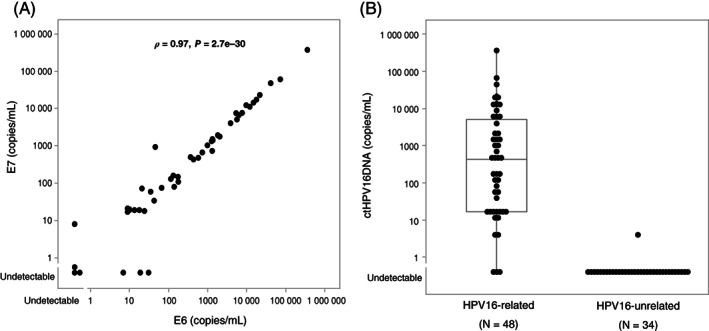
ctHPV16DNA levels in patients with HPV16‐related or HPV16‐unrelated tumors. (A) Correlation in copy number between circulating E6 and E7 DNA in patients with HPV16‐related tumors (N = 48). Spearman's rank correlation was used to evaluate the correlation. (B) ctHPV16DNA levels in patients with HPV16‐related (N = 48) and HPV16‐unrelated (N = 34) tumors. The box boundaries and middle horizontal line indicate the 25th to 75th percentile range and mean, respectively. Solid lines extending above and below the box indicate the range of ctHPV16DNA. ctHPV16DNA, circulating tumor HPV16 DNA; HPV, human papillomavirus

The correlation between the copy numbers of circulating HPV16 E6 and E7 DNA was excellent among patients with HPV16‐related tumors (*ρ* = 0.97, *P* = 2.7e−30), as shown in Figure 1A. Neither E6 nor E7 were detectable in three patients, while either E6 or E7 alone was detectable in four patients. Combination of E6 and E7 favored ctHPV16DNA detection, and resulted in the detection in 45 of 48 patients with HPV16‐related tumors (median, 435 copies/mL; interquartile range, 17‐5016) (Figure 1B). In contrast, ctHPV16DNA was undetectable in all but one of the 34 patients with HPV16‐unrelated tumors (Figure 1B). One patient with an HPV16‐unrelated tumor harbored ctHPV16DNA of 4 copies/mL; however, this is most probably attributable to a false‐positive result that was caused by nonspecific amplification, as detailed in Figure S2. Table 2 summarizes the diagnostic performance of each HPV test. The sensitivity and specificity of ctHPV16DNA were 93% (95% CI = 81‐99) and 97% (95% CI = 84‐100), respectively, for HPV16‐related OPSCC and 100% (95% CI = 54‐100) and 100% (95% CI = 16‐100), respectively, for HPV16‐related SCCUP.

**TABLE 2:** Sensitivity and specificity of HPV tests for HPV‐related OPSCC or SCCUP

		Test	No. of positive	Sensitivity (95% CI)	No. of negative	Specificity (95% CI)
OPSCC	HPV16‐related	ctHPV16DNA	39/42	93% (81‐99)	31/32	97% (84‐100)
		Oral HPV DNA	32/39	82% (66‐92)	29/29	100% (88‐100)
		Oral HPV mRNA	33/39	85% (69‐94)	17/18	94% (73‐100)
	Other high‐risk HPV‐related	Oral HPV DNA	8/11	73% (39‐94)	18/18	100% (81‐100)
		Oral HPV mRNA	8/11	73% (39‐94)	17/18	94% (73‐100)
	Any high‐risk HPV‐related	Oral HPV DNA	40/50	80% (66‐90)	18/18	100% (81‐100)
		Oral HPV mRNA	41/50	82% (69‐91)	17/18	94% (73‐100)
SCCUP	HPV16‐related	ctHPV16DNA	6/6	100% (54‐100)	2/2	100% (16‐100)
		Oral HPV DNA	1/5	20% (1‐72)	1/1	100% (3‐100)
		Oral HPV mRNA	0/5	0% (0‐52)	1/1	100% (3‐100)

Abbreviations: CI, confidence interval; ctHPV16DNA, circulating tumor HPV16 DNA; HPV, human papillomavirus; OPSCC, oropharyngeal squamous cell carcinoma; SCCUP, squamous cell carcinoma of unknown primary.

**FIGURE 2:**
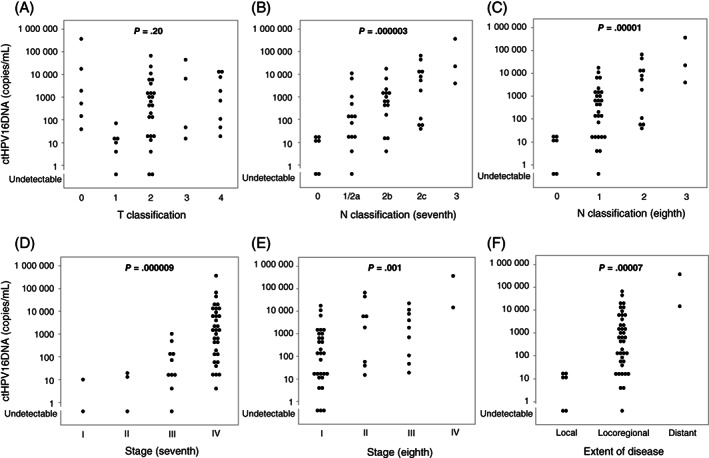
ctHPV16DNA levels as a function of the anatomic extent of disease in patients with HPV16‐related tumors. Trends of ctHPV16DNA levels were examined for (A) T classification, (B) N classification according to the seventh edition of the UICC TNM classification system, (C) N classification according to the eighth edition of the UICC TNM classification system, (D) disease stage according to the seventh edition of the UICC TNM classification system, (E) disease stage according to the eighth edition of the UICC TNM classification system and (F) extent of the disease, using the Jonckheere‐Terpstra test. Local, locoregional and distant represent locally confined disease, locoregionally confined disease and disease with distant metastasis, respectively (F). ctHPV16DNA, circulating tumor HPV16 DNA; HPV, human papillomavirus; UICC, Union for International Cancer Control

As the sensitivity of ctHPV16DNA was equivalent between HPV16‐related OPSCC and HPV16‐related SCCUP, we analyzed the association of ctHPV16DNA with tumor burden after putting these two entities together. Figure 2 shows ctHPV16DNA levels as a function of the anatomic extent of the disease in patients with HPV16‐related tumors. ctHPV16DNA did not exhibit correlation with T classification (*P* = .20, Figure 2A). However, after excluding T0 SCCUP, a significantly increasing trend in ctHPV16DNA levels along with T classification was disclosed (*P* = .007). Importantly, ctHPV16DNA was detectable in all six patients with HPV16‐related SCCUP. Increasing trend of ctHPV16DNA levels was also observed along with the progression of N classification (*P* = .000003 for the seventh edition, Figure 2B and *P* = .00001 for the eighth edition of the UICC TNM classification system, Figure 2C). Likewise, ctHPV16DNA levels increased in proportion to the progression of disease stage regardless of its categorization based on the seventh or eighth edition of the UICC TNM classification system (*P* = .000009 for the seventh edition, Figure 2D and *P* = .001 for the eighth edition, Figure 2E). Moreover, ctHPV16DNA levels also increased along with the progression from locally confined disease to locoregionally confined disease to disease with distant metastasis (*P* = .00007, Figure 2F). Patient characteristics that were unrelated to the extent of the disease, such as primary tumor subsite, as well as the patient's smoking history, age and sex, did not affect ctHPV16DNA levels (Figure S3).

**FIGURE 3:**
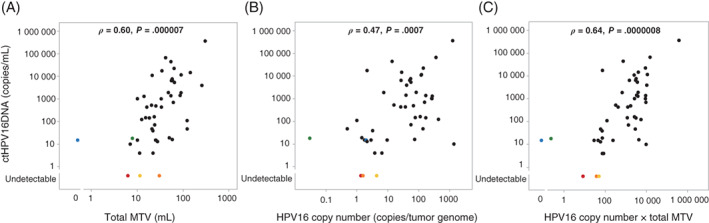
Correlation of ctHPV16DNA levels with (A) total MTV, (B) HPV16 copy number per tumor genome and (C) the product of total MTV and HPV16 copy number per tumor genome in patients with HPV16‐related tumors. Red, orange and yellow dots represent three individual patients in whom ctHPV16DNA was undetectable. Blue and green dots represent two individual patients in whom ctHPV16DNA was detectable, although the product of total MTV and HPV16 copy number per tumor genome was zero and minimal, respectively. Spearman's rank correlation was used to evaluate the correlation. ctHPV16DNA, circulating tumor HPV16 DNA; HPV, human papillomavirus; MTV, metabolic tumor volume [Color figure can be viewed at wileyonlinelibrary.com]

Considering that we previously reported the significant correlation of MTV with the anatomic extent of the disease,^14^, ^15^ we examined whether ctHPV16 DNA levels would show correlation with MTV. We observed a moderate correlation between ctHPV16DNA levels and total MTV (*ρ* = 0.60, *P* = .000007) (Figure 3A). As HPV copy number per tumor genome varies widely according to each individual tumor,^16^ we also examined the correlation between ctHPV16DNA levels and HPV16 copy number per tumor genome and observed a moderate correlation between the two (*ρ* = 0.47, *P* = .0007) (Figure 3B). We further examined the correlation between ctHPV16DNA levels and the product of HPV16 copy number per tumor genome and total MTV. This is because total MTV would be directly proportional to the number of cancer cells. We found that the correlation was improved, although it remained moderate (*ρ* = 0.64, *P* = .0000008) (Figure 3C), suggesting that while the product was crucial for determining ctHPV16DNA levels, certain unspecified factors other than the product might affect ctHPV16DNA levels.

Specifically, ctHPV16DNA was undetectable in three patients with OPSCC classified as T1N0M0, T2N0M0 and T2N1M0 (the seventh edition of the UICC TNM classification system). One patient with T1N0M0 disease presented with an HPV16 copy number per tumor genome of 1.4 and a total MTV of 6.2 mL, which yielded a small product of 8.68, thus providing an explanatory approach for the observation of undetectable ctHPV16DNA levels. In contrast, the other two patients presented with higher HPV16 copy number per tumor genome (4.4 and 1.6, respectively) and higher total MTVs (11.4 and 30.2 mL, respectively), yielding products of 50.16 and 48.32, respectively. Considering that ctHPV16DNA was detectable in other patients with products of the same levels, it was suggested that ctHPV16DNA was undetectable due to certain unspecified factors in these two patients. Conversely, ctHPV16DNA was detectable in two patients whose product of HPV16 copy number per tumor genome and total MTV was minimal (0.23) or zero. One patient with T2N1M0 OPSCC (the seventh edition of the UICC TNM classification system) presented with an HPV16 copy number per tumor genome of 0.03, and a total MTV of 7.8 mL, yielding a minimal product. The proportion of tumor cells to nontumor cells in his biopsy specimen was most likely small, which might have resulted in seemingly lower HPV copy number per tumor genome than the actual number. The other patient harbored locally advanced OPSCC classified as T1N2bM0 according to the seventh edition of the UICC TNM classification system, but the total MTV was immeasurably small because SUVmax of the tumor was 2.4.

### 3.3 Performance of oral HPV DNA

Table S3 shows the concordance in genotypes between tumor HPV DNA and oral HPV DNA in patients with HPV‐related tumors. Oral HPV DNA of any genotype was detectable in 42 of the 55 patients, while oral HPV DNA, the genotype of which was harmonious with that of tumor HPV DNA, was detectable in 41 patients. Oral HPV DNA, the genotype of which was different from that of tumor HPV DNA, was detected in five patients with HPV‐related tumors. None of the 19 patients with HPV‐unrelated tumors showed positive results for oral HPV DNA of any low‐risk or high‐risk genotype. As shown in Table 2, the sensitivity and specificity of oral HPV DNA for HPV16‐related OPSCC were 82% (95% CI = 66‐92) and 100% (95% CI = 88‐100), respectively. As for OPSCC that was related to high‐risk HPV except for HPV16, the sensitivity and specificity of oral HPV DNA were 73% (95% CI = 39‐94) and 100% (95% CI = 81‐100), respectively. As GENOSEARCH HPV31 does not include HPV69 within its detection range, oral HPV69 DNA was undetectable in both patients with HPV69‐related OPSCC (Table S3). Collectively, the sensitivity and specificity of oral HPV DNA for any high‐risk HPV related OPSCC were 80% (95% CI = 66‐90) and 100% (95% CI = 81‐100), respectively. In contrast to the relatively high sensitivity of oral HPV DNA for HPV16‐related OPSCC, the sensitivity of oral HPV DNA for HPV16‐related SCCUP was poor. The sensitivity and specificity of oral HPV DNA for HPV16‐related SCCUP were 20% (95% CI = 1‐72) and 100% (95% CI = 3‐100), respectively. Of interest, three of four patients with HPV16‐related SCCUP who showed negative oral HPV DNA test results underwent thorough inspection of the oropharynx, including tonsillectomy, under general anesthesia, and one patient had the p16‐positive/HPV16 DNA‐positive occult primary proved at the base of the tongue. Unfortunately, however, one patient with HPV16‐related SCCUP who showed a positive oral HPV16 DNA test result did not undergo such an inspection prior to treatment, and the primary tumor remained unknown.

As primary tumor status, OPSCC or SCCUP, had a major impact on the sensitivity of oral HPV DNA, we focused on patients with HPV‐related OPSCC to assess the association of oral HPV DNA with tumor burden. Table 3 shows oral HPV DNA‐positivity according to the characteristics observed in patients with HPV16‐related OPSCC. There was no significant difference between subsites among patients with OPSCC (*P* = .59). Neither T classification, N classification nor disease stage showed correlation with oral HPV DNA‐positivity, irrespective of the seventh and eighth editions of the UICC TNM classification system. This suggests that oral HPV DNA‐positivity was independent of the anatomic extent of the disease in patients with HPV16‐related OPSCC. Additionally, neither total MTV nor HPV16 copy number per tumor genome exhibited correlation with oral HPV DNA‐positivity in patients with HPV16‐related OPSCC. Oral HPV DNA‐positivity according to the characteristics observed in patients with any high‐risk HPV‐related OPSCC is shown in Table S4.

**TABLE 3:** Oral HPV test positivity according to characteristics in patients with HPV16‐related OPSCC

Characteristics	Level	No.	Oral HPV DNA		Oral HPV mRNA	
			No. of positive (%, 95% CI)	*P* value	No. of positive (%, 95% CI)	*P* value
Age	<65	14	12 (86%, 57‐98)	1.00	13 (93%, 66‐100)	.39
	≥65	25	20 (80%, 59‐93)		20 (80%, 59‐93)	
Sex	Male	30	24 (80%, 61‐92)	1.00	25 (83%, 65‐94)	1.00
	Female	9	8 (89%, 52‐100)		8 (89%, 52‐100)	
Pack‐years of smoking	<10	13	12 (92%, 64‐100)	.39	12 (92%, 64‐100)	.64
	≥10	26	20 (77%, 56‐91)		21 (81%, 61‐93)	
Primary site	Lateral wall	31	26 (84%, 66‐95)	.59^a^	27 (87%, 70‐96)	.30^a^
	Anterior wall	7	5 (71%, 29‐96)		5 (71%, 29‐96)	
	Posterior wall	1	1 (100%, 3‐100)		1 (100%, 3‐100)	
T classification	1, 2	30	25 (83%, 65‐94)	.65	25 (83%, 65‐94)	1.00
	3, 4	9	7 (78%, 40‐97)		8 (89%, 52‐100)	
N classification^b^	0, 1, 2a	18	17 (94%, 73‐100)	.10	17 (94%, 73‐100)	.19
	2b, 2c, 3	21	15 (71%, 48‐89)		16 (76%, 53‐92)	
N classification^c^	0, 1	31	27 (87%, 70‐96)	.14	27 (87%, 70‐96)	.58
	2, 3	8	5 (63%, 24‐91)		6 (75%, 35‐97)	
Stage^b^	I, II	5	5 (100%, 48‐100)	.56	5 (100%, 48‐100)	.57
	III, IV	34	27 (79%, 62‐91)		28 (82%, 65‐93)	
Stage^c^	I, II	32	27 (84%, 67‐95)	.59	27 (84%, 67‐95)	1.00
	III, IV	7	5 (71%, 29‐96)		6 (86%, 42‐100)	
Total MTV (mL)	<25	17	13 (76%, 50‐93)	.68	13 (76%, 50‐93)	.37
	≥25	22	19 (86%, 65‐97)		20 (91%, 71‐99)	
HPV16 copy number (copies/tumor genome)	<40	19	15 (79%, 54‐94)	.69	16 (84%, 60‐97)	1.00
	≥40	20	17 (85%, 62‐97)		17 (85%, 62‐97)	

*Note*: Statistical analyses were made using Fisher's exact test.

Abbreviations: CI, confidence interval; HPV, human papillomavirus; MTV, metabolic tumor volume; OPSCC, oropharyngeal squamous cell carcinoma; UICC, Union for International Cancer Control.

^a^ Difference was estimated between lateral wall and anterior wall.

^b^ According to the seventh edition of UICC TNM classification system.

^c^ According to the eighth edition of UICC TNM classification system.

### 3.4 Performance of oral HPV mRNA

Oral HPV mRNA test showed results that were similar to those of the oral HPV DNA test. Oral HPV mRNA was detectable in 41 of the 50 patients with any high‐risk HPV‐related OPSCC and was undetectable in 17 of the 18 patients with HPV‐unrelated OPSCC, indicating that the sensitivity and specificity of oral HPV mRNA for any high‐risk HPV‐related OPSCC were 82% (95% CI = 69‐91) and 94% (95% CI = 73‐100), respectively (Table 2). One patient with HPV‐unrelated OPSCC was positive for oral HPV mRNA; however, its clinical relevance remains unknown. Of note, 41 of the 50 patients with any high‐risk HPV‐related OPSCC were positive for oral HPV DNA and/or mRNA. Forty (98%) of the 41 patients were positive for both, indicating a high concordance between the two oral HPV test results. When restricted to HPV16‐related OPSCC, the sensitivity and specificity of oral HPV mRNA were 85% (95% CI = 69‐94) and 94% (95% CI = 73‐100), respectively. The combination with ctHPV16DNA increased the sensitivity up to 100% (95% CI = 92‐100). Alternatively, when restricted to OPSCC that was related to high‐risk HPV except for HPV16, the sensitivity and specificity of oral HPV mRNA were 73% (95% CI = 39‐94) and 94% (95% CI = 73‐100), respectively. Both patients with HPV69‐related OPSCC showed negative results for oral HPV mRNA test because HPV69 is not included in genotypes that Aptima is capable of detecting. Similarly to oral HPV DNA, oral HPV mRNA exhibited a poor sensitivity for HPV16‐related SCCUP. The sensitivity and specificity of oral HPV mRNA for HPV16‐related SCCUP were 0% (95% CI = 0‐52) and 100% (95% CI = 3‐100), respectively, indicating that both oral HPV DNA and mRNA do not yield fruitful results in the identification of patients with HPV16‐related SCCUP. As with oral HPV DNA, oral HPV mRNA showed no difference in positivity between subsites among patients with HPV16‐related OPSCC (*P* = .30, Table 3). Moreover, there was no correlation between oral HPV mRNA‐positivity and either the anatomic extent of the disease, total MTV or HPV16 copy number per tumor genome in patients with HPV16‐related OPSCC. Similar results were obtained for patients with any high‐risk HPV‐related OPSCC (Table S4).

Collectively, the specificity was high across all three biomarkers, whereas the sensitivity was divergent between them. The sensitivity of ctHPV16DNA for HPV16‐related OPSCC was higher than that of oral HPV DNA or oral HPV mRNA. However, the difference was statistically significant only for HPV16‐related SCCUP between ctHPV16DNA and oral HPV mRNA. This is because the 95% CI of the sensitivity for HPV16‐related OPSCC overlapped between ctHPV16DNA and two oral biomarkers most probably due to the relatively small sample size. Likewise, such an overlap was also observed for HPV16‐related SCCUP between ctHPV16DNA and oral HPV DNA. ctHPV16DNA well correlated with the extent of tumor burden in patients with HPV16‐related tumors, whereas oral HPV DNA or oral HPV mRNA did not.

## 4 DISCUSSION

The observed sensitivity (93%) of ctHPV16DNA for HPV16‐related OPSCC seemed higher than, or equivalent to, that reported previously, which ranged from 71% to 96%.^17^, ^18^, ^19^ There was a difference in patient characteristics between studies, especially in terms of the extent of the disease. Damerla et al reported the highest sensitivity of 96%, although the vast majority of examined patients presented with Stage IV (the seventh edition of the UICC TNM classification system) disease and none presented with T1‐T2N0 disease, which could have favored an increase in sensitivity.^19^ These previous reports also quantified ctHPV16DNA levels using individually optimized in‐house ddPCR, although either E6 or E7 alone was analyzed. We analyzed both E6 and E7 and defined ctHPV16DNA as the average of the two. We found an excellent correlation between E6 and E7 copy numbers, while four (8%) patients were positive only for either E6 or E7 alone, indicating that the dual measurement of E6 and E7 demonstrated an advantage over the single measurement regarding sensitivity. As HPV16 DNA‐positivity for either E6 or E7 alone tended to occur when ctHPV16DNA levels were low, the dual measurement would enhance ctHPV16DNA detection, especially in patients with low tumor burden and/or with tumors of low HPV16 copy number per tumor genome. Trivial differences in experimental procedures, including the amount of plasma used for ddPCR, might have also contributed to the difference in sensitivity. Therefore, the ddPCR procedures for quantifying ctHPV16DNA should be optimized universally.

We observed a significant correlation between ctHPV16DNA levels and the product of HPV16 copy number per tumor genome and total MTV in patients with HPV16‐related tumors. Undoubtedly, ctHPVDNA is likely undetectable in patients with tumors with low HPV copy number per tumor genome, especially when the tumor volume is small. In this regard, we should recognize the pitfalls of ctHPVDNA‐guided surveillance for recurrent disease after definitive treatment. Chera et al reported that longitudinal monitoring of ctHPVDNA would facilitate early identification of recurrent disease in patients with HPV‐related OPSCC.^20^ Nonetheless, ctHPVDNA monitoring would probably not enable an earlier diagnosis of disease recurrence compared with conventional imagings in patients with tumors of low HPV copy number per tumor genome.

We found previously that whenever SCCUP with metastatic node was HPV DNA‐positive, it was also p16‐positive.^9^ Accordingly, we defined SCCUP harboring HPV DNA‐positive metastatic node as HPV‐related even when tumor p16 status was unknown. Some would argue that HPV DNA‐positive metastatic node alone is insufficient to define SCCUP as HPV‐related and that the evidence of p16 overexpression in tumor cells is indispensable to ensure HPV‐driven transformation. This is because the contamination of sample or laboratory equipment with HPV DNA can happen, which will result in false‐positivity of HPV DNA. However, we found that all six SCCUP patients with HPV16 DNA‐positive metastatic nodes were also positive for ctHPV16DNA in plasma without exception, while tumor p16 status was positive and unknown in two and four of the six patients, respectively. Considering the excellent positive predictive value of ctHPV16DNA for “truly” HPV16‐related OPSCC that 39 (98%) of 40 positive test results were true‐positive (Table 2), the p16‐unknown tumors of the four patients were most probably p16‐positive and thus “truly” HPV16‐related. Of course, the possibility cannot be ruled out completely that both HPV DNA tests for metastatic node and plasma yield false‐positive results. Nonetheless, we believe that node‐positive SCCUP is “truly” HPV‐related when both HPV DNA in metastatic node and ctHPVDNA in plasma are detectable and when there is no evidence of HPV‐related cervical or anogenital cancer to which ctHPVDNA is attributable. Currently, an invasive approach, such as open biopsy of the neck for assessing tumor p16 status, is required to determine whether node‐positive SCCUP is HPV‐related or ‐unrelated. Such an approach might be no longer necessary and might be replaced with less invasive approaches, such as fine needle aspiration and liquid biopsy for assessing HPV DNA status in metastatic nodes and ctHPVDNA in plasma, respectively.

Oral HPV DNA has been previously examined in patients with HPV‐related OPSCC using commercially available assays, such as DNA ELISA kit HPV SPF10, version 1 (Labo Bio‐medical Products B.V., Rijswijk, Netherlands),^21^ Cobas HPV Test (Roche)^21^ and Roche Linear Array HPV Genotyping Test (Roche).^22^, ^23^ Sensitivity ranged from 49% to 84% in these studies. We used GENOSEARCH HPV31 and determined a sensitivity of 80% for HPV‐related OPSCC. These results indicate that the sensitivity of oral HPV DNA is unsatisfactory, regardless of the HPV assay used. The sensitivity of oral HPV DNA may be improved if analysis is conducted using ddPCR, although this has never been reported. However, Hanna et al examined oral HPV DNA levels in patients with recurrent or persistent OPSCC using ddPCR, demonstrating a sensitivity of 87%.^24^ On the other hand, we observed an excellent specificity of oral HPV DNA (100%). However, this might be a result by chance because the prevalence of oral high‐risk HPV infection in healthy Japanese individuals is 4.4%.^25^

D'Souza et al previously reported the efficiency of oral HPV mRNA in detecting HPV‐related OPSCC.^21^ They also used Aptima as the HPV mRNA assay and reported a sensitivity of 23%, which was considerably poorer compared with our finding: a sensitivity of 82% for HPV‐related OPSCC. It is difficult to propose a plausible explanation for the difference in sensitivity between the two reports. Of note, however, the samples analyzed by D'Souza et al. seem to have been stored for a long period, while we analyzed freshly prepared samples.

It is reasonable to assume that the number of tumor cells in oral samples that are exfoliated from primary tumor during gargling would affect the detection efficiency for oral HPV DNA and oral HPV mRNA. Oral HPV DNA and oral HPV mRNA are likely detectable in patients with HPV‐related OPSCC, while these oral biomarkers are hardly detectable in patients with HPV‐related SCCUP. Actually, our findings substantiated the assumption: the sensitivity of oral HPV DNA was 82% for HPV16‐related OPSCC, but only 20% for HPV16‐related SCCUP. Likewise, the sensitivity of oral HPV mRNA was 85% for HPV16‐related OPSCC, but 0% for HPV16‐related SCCUP. Moreover, considering that neither oral HPV DNA nor oral HPV mRNA detection showed any correlation with the extent of the local disease, such as T classification, in patients with HPV‐related OPSCC, other unspecified factors seem crucial for exfoliating cells from tumor. We speculate that patients who could not gargle well likely presented with negative results in the oral HPV tests. While the skill of oral gargling cannot be easily and objectively evaluated, the number of exfoliated cells may be considered a surrogate measure of oral gargling efficiency. Unfortunately, however, we did not determine the number of exfoliated cells.

HPV‐related OPSCC is a slow‐growing entity and ctHPV16DNA exhibits satisfactorily high sensitivity and specificity in detecting HPV‐related OPSCC, suggesting the utility of ctHPV16DNA‐guided screening for HPV16‐related OPSCC. However, considering a low lifetime risk^26^ and favorable prognosis^27^ of HPV‐related OPSCC and a lack of identifiable precursors, it is unlikely that population‐based screening would contribute toward a reduction in mortality. Thus, such a screening would not be warranted in the general population. Meanwhile, screening among subpopulations that have an increased risk of developing HPV‐related OPSCC, such as men who currently smoke and have a higher number of oral sex partners in their lifetime,^26^ may prove to be useful. It should be noted that HPV‐related cancer is not limited to OPSCC. Cervical and anogenital cancers are also driven by HPV infection. Of the 14 million new cancer cases reported globally in 2012, 640 000 (4.6%) were attributable to HPV.^28^, ^29^ Considering that ctHPVDNA serves as a biomarker for HPV‐related cancers, irrespective of the anatomic site,^30^, ^31^ ctHPVDNA‐guided population‐based screening extended to all HPV‐related cancers may be appropriate.

HPV DNA detection is relevant to HPV infection but not always to HPV‐driven transformation, whereas HPV mRNA detection is relevant to HPV‐driven transformation.^32^ Oral HPV mRNA is detectable in a small subset of cancer‐free individuals with positive results for oral HPV DNA tests, whereas it is never detectable in those presenting with negative results for oral HPV DNA tests.^33^ These findings suggest that oral HPV mRNA is suitable for HPV‐related OPSCC screening. However, considering the moderate sensitivity (82%) of oral HPV mRNA in detecting HPV‐related OPSCC, oral HPV mRNA‐guided screening will often overlook true‐positive cases. Thus, such a screening is not warranted even in individuals who are at an increased risk of developing HPV‐related OPSCC. As the combination of oral HPV mRNA with ctHP16VDNA yielded an excellent sensitivity (100%), it may facilitate the screening of the high‐risk subpopulations.

The performance of HPV16 E6 serum antibody for HPV16‐related OPSCC has been previously explored in several studies that have reported high sensitivity (88%‐96%) and specificity (96%‐98%).^21^, ^34^, ^35^ Particularly, D'Souza et al reported that the detection of HPV16 E6 serum antibody yielded superior results to oral HPV tests.^21^ However, we favor ctHPV16DNA over the HPV16 E6 serum antibody. This is because ctHPV16DNA is a quantitative biomarker that has a potential for improving the management of HPV‐related OPSCC, while HPV16 E6 serum antibody is rather qualitative. For example, Chera et al observed that the dynamics of ctHPV16DNA levels during chemoradiotherapy were predictive of the response.^17^ Likewise, we also found that the dynamics during induction chemotherapy were predictive of the response (unpublished findings). Moreover, ctHPV16DNA detection was shown to precede CT and FDG‐PET/CT in identifying persistent or recurrent disease after definitive treatment.^13^, ^20^ The ctHPV16DNA‐guided molecular response may soon replace the CT‐ and FDG‐PET/CT‐guided anatomic and metabolic response, respectively, for applications in monitoring the treatment efficacy for HPV‐related OPSCC.

This study has several limitations. The sensitivity of ctHPV16DNA for detecting HPV16‐related OPSCC was higher than that of oral HPV DNA or oral HPV mRNA. However, the advantage of ctHPV16DNA over oral HPV DNA or oral HPV mRNA should be interpreted with caution because the difference in sensitivity was not statistically significant. Moreover, the sample size of patients with SCCUP was especially small. Thus, the results for SCCUP were preliminary. ctHPVDNA analysis was performed using our in‐house ddPCR system and was limited to HPV16. The development of a commercially available multiplex ddPCR assay that widely covers high‐risk HPV genotypes is required to familiarize ctHPVDNA‐guided management of HPV‐related OPSCC. Oral HPV DNA was measured using GENOSEARCH HPV31, a commercially available HPV DNA assay, which is not globally distributed, although it is common in Japan. Neither oral HPV DNA nor mRNA was subjected to quantitative measurements, and these biomarkers were not considered as continuous variables.

In conclusion, ctHPV16DNA is a potentially promising biomarker for the detection of HPV16‐related OPSCC. Further studies are required to establish the usefulness of ctHPV16DNA in distinguishing HPV16‐related SCCUP from HPV16‐unrelated SCCUP.

## CONFLICT OF INTEREST

The authors declare no conflicts of interest.

## ETHICS STATEMENT

The study received ethical approval from the institutional review boards of Osaka University. Written informed consent was obtained from all patients.

## Supporting information


**Appendix S1**: Supplementary Information

## Data Availability

Further details and other data that support the findings of this study are available from the corresponding author upon request.
