# Supplementary material for: Performance of oral HPV DNA, oral HPV mRNA and circulating tumor HPV DNA in the detection of HPV‐related oropharyngeal cancer and cancer of unknown primary
Source: Int J Cancer. 2021 Sep 20;150(1):174–86. doi: 10.1002/ijc.33798 (PMC9290341; doi:10.1002/ijc.33798)
Supplement: Supplementary file 1 — Appendix S1: Supplementary Information [file IJC-150-174-s001.pdf]

Performance of Oral HPV DNA, Oral HPV mRNA, and Circulating Tumor HPV DNA in the Detection of HPV-Related Oropharyngeal Cancer and Cancer of Unknown Primary

Hidenori Tanaka, Motoyuki Suzuki, Norihiko Takemoto, Takahito Fukusumi,
Hirotaka Eguchi, Erina Takai, Haruka Kanai, Mitsuaki Tatsumi, Masafumi Horie,
Yukinori Takenaka, Shinichi Yachida, and Hidenori Inohara

Supplementary material

Table of contents:

Supplementary Table S1. Baseline characteristics of patients with other high-risk HPV-related OPSCC or SCCUP.

Supplementary Table S2. Baseline characteristics of patients with HPV-unrelated OPSCC or SCCUP.

Supplementary Table S3. Concordance in genotype between tumor HPV and oral HPV in patients with HPV-related OPSCC or SCCUP.

Supplementary Table S4. Oral HPV test positivity according to characteristics in patients with any high-risk HPV-related OPSCC.

Supplementary Figure S1. Flowchart of the study population and sample collection for patients with OPSCC and patients with SCCUP.

Supplementary Figure S2. Two-channel analysis of FAM and HEX for ddPCR.

Supplementary Figure S3. Correlation of ctHPV16DNA levels with characteristics unrelated to the extent of disease in patients with HPV16-related tumor.

Supplementary Table S1. Baseline characteristics of patients with other high-risk HPV-related OPSCC or SCCUP

	**Other high-risk HPV-related OPSCC**	**Other high-risk HPV-related SCCUP**
	**Oral and blood sample, N = 11**	**Blood sample, N = 1**
**Sex**		
Male	8	1
Female	3	0
**Age**		
Median	75	66
Range	63–90	-
**Smoking history**		
< 10 pack-years	5	0
≥ 10 pack-years	6	1
**p16 IHC / HPV DNA status**		
p16-positive / other high-risk HPV-positive	11	1
p16-unknown / other high-risk HPV-positive	0	0
**Primary subsite**		
Lateral wall	7	-
Anterior wall	3	-
Posterior wall	1	-
Unknown	-	1
**T classification**		
T0/T1/T2/T3/T4	-/2/5/2/2	1/-/-/-/-

**N classification***		
N0/N1/N2a/N2b/N2c/N3	2/0/2/4/2/1	0/0/0/1/0/0
**N classification†**		
N0/N1/N2/N3	2/6/2/1	0/1/0/0
**M classification**		
M0/M1	10/1	1/0
**Stage***		
I/II/III/IV	0/2/1/8	0/0/0/1
**Stage†**		
I/II/III/IV	7/1/2/1	1/0/0/0

* According to the 7th edition of UICC TNM classification system.

† According to the 8th edition of UICC TNM classification system.

Abbreviations: HPV, human papillomavirus; IHC, immunohistochemistry; OPSCC, oropharyngeal squamous cell carcinoma; SCCUP, squamous cell carcinoma of unknown primary; UICC, Union for International Cancer Control.

Supplementary Table S2. Baseline characteristics of patients with HPV-unrelated OPSCC or SCCUP

	HPV-unrelated OPSCC		HPV-unrelated SCCUP
	**Oral sample, N = 18**	**Blood sample, N = 21**	**Oral and blood sample, N = 1**
**Sex**			
Male	16	18	1
Female	2	3	0
**Age**			
Median	70	70	81
Range	39–82	39–82	-
**Smoking history**			
< 10 pack-years	1	3	0
≥ 10 pack-years	17	18	1
**p16 IHC / HPV DNA status**			
p16-positive / HPV-negative	6	7	0
p16-negative / HPV-negative	12	14	1
p16-unknown / HPV-negative	0	0	0
**Primary subsite**			
Lateral wall	10	12	-
Anterior wall	5	6	-
Posterior wall	2	2	-
Superior wall	1	1	-
Unknown	-	-	1

**T classification**			
T0/T1/T2/T3/T4	-/2/7/4/5	-/3/8/4/6	1/-/-/-/-
**N classification***			
N0/N1/N2a/N2b/N2c/N3	2/3/0/7/6/0	4/3/0/8/6/0	0/0/0/0/0/1
**M classification**			
M0/M1	18/0	21/0	1/0
**Stage***			
I/II/III/IV	1/1/3/13	2/1/3/15	0/0/0/1

*According to the 7th edition of UICC TNM classification system.

Abbreviations: HPV, human papillomavirus; IHC, immunohistochemistry; OPSCC, oropharyngeal squamous cell carcinoma; SCCUP, squamous cell carcinoma of unknown primary; UICC, Union for International Cancer Control.

Supplementary Table S3. Concordance in genotype between tumor HPV and oral HPV in patients with HPV-related OPSCC or SCCUP

	Tumor HPV genotype	No. of patients	Oral HPV Genotype*	No. of patients
**OPSCC (N = 50)**	HPV16	39	HPV16	28
			HPV16, 59	1
			HPV16, 66	1
			HPV16, 82	1
			HPV16, 6, 11, 39, 59, 82	1
			Undetectable	7
	HPV18	2	HPV18	2
	HPV31	1	HPV31	1
	HPV33	1	HPV33	1
	HPV35	3	HPV35	3
	HPV56	1	Undetectable	1
	HPV58	1	HPV58	1
	HPV69[†]	2	HPV71	1
			Undetectable	1
**SCCUP (N = 5)**	HPV16	5	HPV16	1
			Undetectable	4

* Low-risk HPV genotypes are underlined.

† HPV69 is not covered by GENOSERCH HPV31 and this is undetectable as oral HPV.

Abbreviation: HPV, human papillomavirus; OPSCC, oropharyngeal squamous cell carcinoma; SCCUP, squamous cell carcinoma of unknown primary.

Supplementary Table S4. Oral HPV test positivity according to characteristics in patients with any high-risk HPV-related OPSCC

			Oral HPV DNA			Oral HPV mRNA		
			No. of positive			No. of positive		
**Characteristics**	**Level**	**No.**	**(%, 95%CI)**		***P* value**	**(%, 95%CI)**		***P* value**
Age	< 65	15	13	(87%, 60–98)	0.70	14	(93%, 68–100)	0.25
	≥ 65	35	27	(77%, 60–90)		27	(77%, 60–90)	
Sex	Male	38	30	(79%, 63–90)	1.00	31	(82%, 66–92)	1.00
	Female	12	10	(83%, 52–98)		10	(83%, 52–98)	
Pack-years of smoking	< 10	18	16	(89%, 65–99)	0.29	16	(89%, 65–99)	0.46
	≥ 10	32	24	(75%, 57–89)		25	(78%, 60–91)	
HPV DNA	HPV16	39	32	(82%, 66–92)	0.67	33	(85%, 69–94)	0.39
	Other high-risk HPV	11	8	(73%, 39–94)		8	(73%, 39–94)	
Primary site	Lateral wall	38	31	(82%, 66–92)	0.41*	32	(84%, 69–94)	0.37*
	Anterior wall	10	7	(70%, 35–93)		7	(70%, 35–93)	
	Posterior wall	2	2	(100%, 16–100)		2	(100%, 16–100)	
T classification	1, 2	37	29	(78%, 62–90)	1.00	29	(78%, 62–90)	0.41
	3, 4	13	11	(85%, 55–98)		12	(92%, 64–100)	
N classification[†]	0, 1, 2a	22	20	(91%, 71–99)	0.15	20	(91%, 71–99)	0.27
	2b, 2c, 3	28	20	(71%, 51–87)		21	(75%, 55–89)	
N classification[‡]	0, 1	39	32	(82%, 66–92)	0.67	32	(82%, 66–92)	1.00
	2, 3	11	8	(73%, 39–94)		9	(82%, 48–98)	
Stage[†]	I, II,	7	6	(86%, 42–100)	1.00	6	(86%, 42–100)	1.00

	III, IV	43	34	(79%, 64–90)		35	(81%, 67–92)	
Stage‡	I, II	40	32	(80%, 64–91)	1.00	32	(80%, 64–91)	0.67
	III, IV	10	8	(80%, 44–97)		9	(90%, 56–100)	

Statistical analyses were made using Fisher's exact test.

Abbreviations: CI, confidence interval; HPV, human papillomavirus; MTV, metabolic tumor volume; OPSCC, oropharyngeal squamous cell carcinoma; UICC, Union for International Cancer Control.

* Difference was estimated between lateral wall and anterior wall.

† According to the 7th edition of UICC TNM classification system.

‡ According to the 8th edition of UICC TNM classification system.

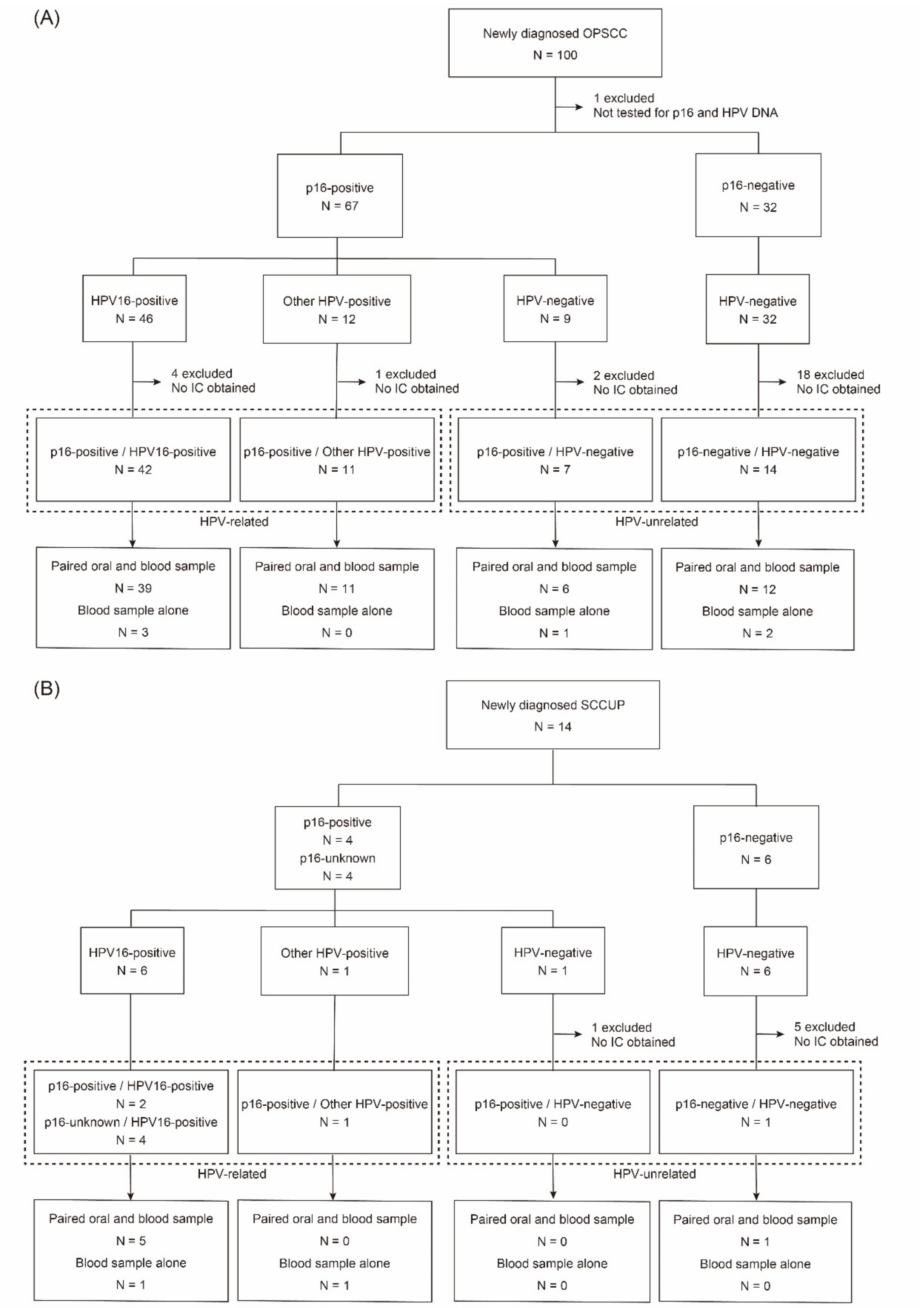

Supplementary Figure S1. Flowchart of the study population and sample collection for patients with OPSCC (A) and patients with SCCUP (B). HPV, human papillomavirus; HR, high-risk; IC, informed consent; OPSCC, oropharyngeal squamous cell carcinoma; SCCUP, squamous cell carcinoma of unknown primary.

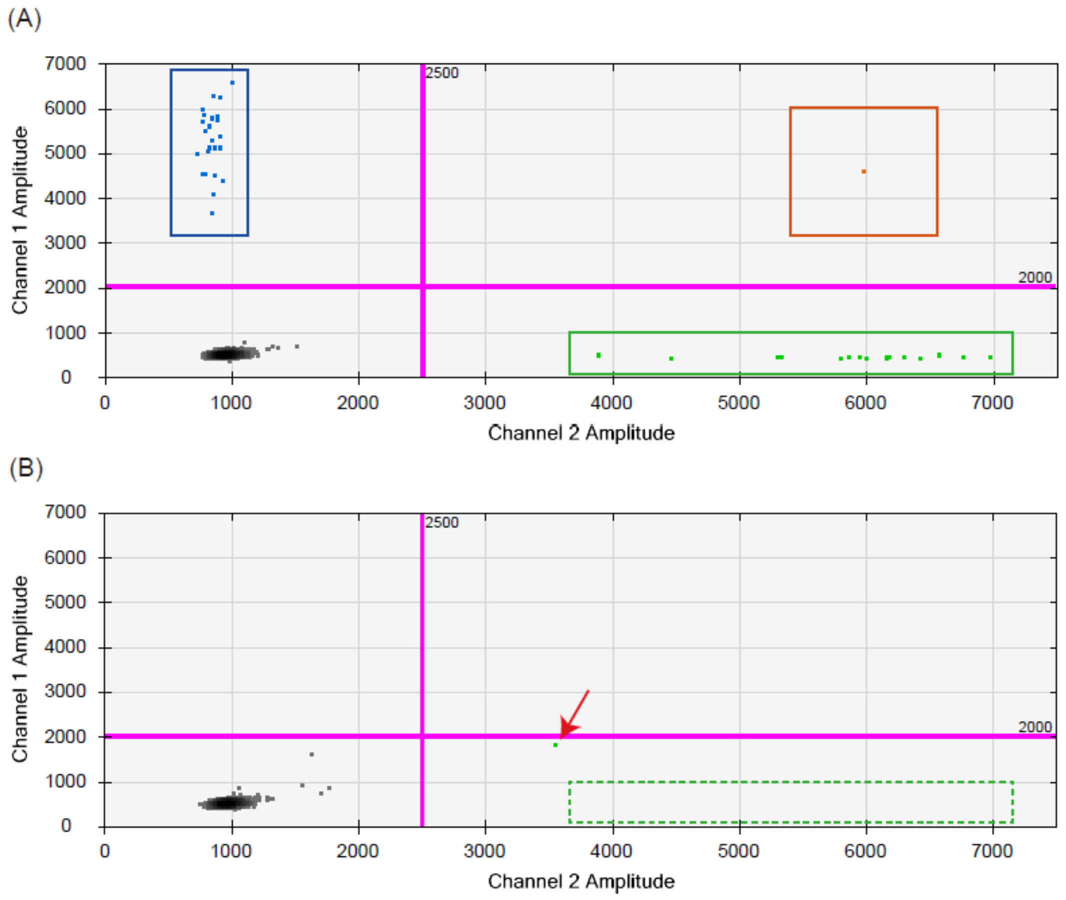

Supplementary Figure S2. Two-channel analysis of FAM and HEX for ddPCR. The absorbance values of the droplets are shown for (A) a representative patient with a HV16-related tumor in whom ctHPV16DNA was detectable and for (B) a patient with a HPV16-unrelated tumor in whom ctHPV16DNA was detectable. The horizontal and vertical axes represent the absorbance of HEX-labeled E6 and FAM-labeled E7, respectively. In patient (A), E6- and E7-positive droplets are enclosed in a green and blue solid frame, respectively. Droplets that are both E6- and E7-positive are enclosed in an orange solid frame. In patient (B), neither E6- nor E7-positive droplets were observed, except for one droplet that has been indicated by using an arrow. This droplet shows the absorbance of HEX-labeled E6 exceeding the threshold; however, it is localized differently from E6-positive droplets that are observed in patient (A), demonstrated in a green-dotted frame. It is suggested that this droplet is formed by non-specific amplification; thus, ctHPV16DNA detection is a false-positive result. ctHPV16DNA, circulating tumor HPV16 DNA; ddPCR, droplet digital PCR; FAM, 6-carboxyfluorescein; HEX, 6-carboxy-2,4,4,5,7,7-hexachlorofluorescein; HPV, human papillomavirus.

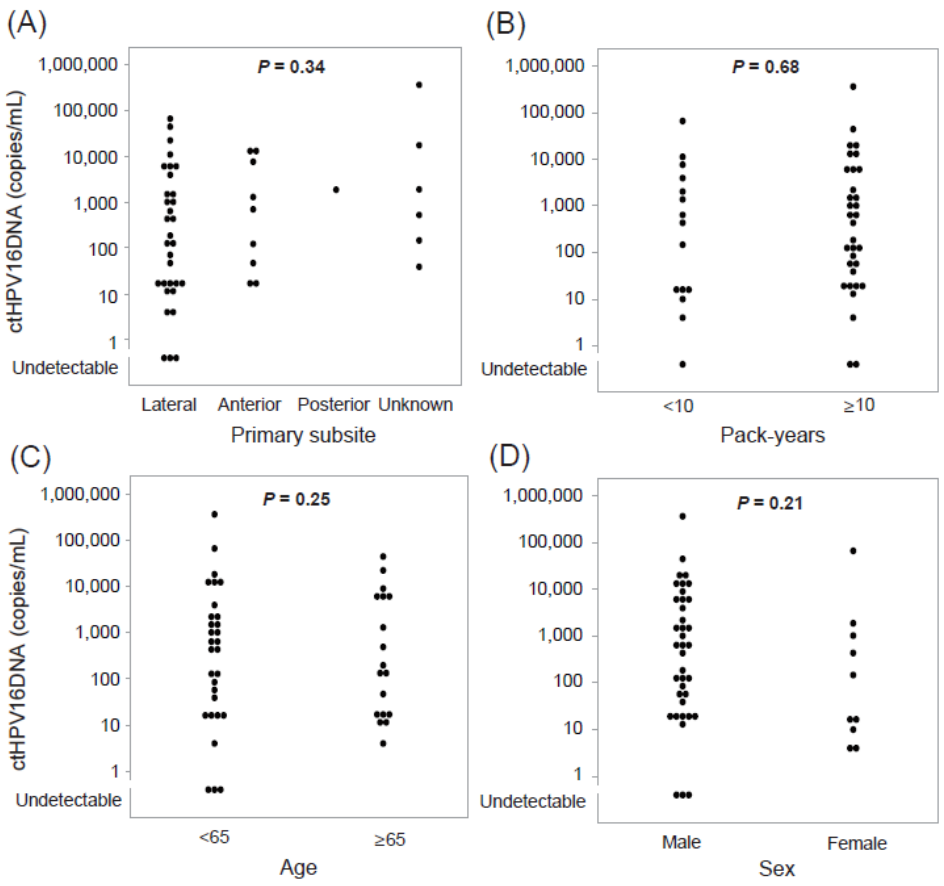

Supplementary Figure S3. Correlation of ctHPV16DNA levels with characteristics unrelated to the extent of disease in patients with HPV16-related tumors. The correlation of ctHPV16 DNA levels with (A) primary subsite was examined, using Kruskal-Wallis test, while the correlations of ctHPV16DNA levels with (B) smoking history (pack-years), (C) age, and (D) sex were examined, using exact Wilcoxon rank-sum test. ctHPV16DNA, circulating tumor HPV16 DNA; HPV, human papillomavirus.
